# Three classes of propofol binding sites on GABA_A_ receptors

**DOI:** 10.1016/j.jbc.2024.107778

**Published:** 2024-09-11

**Authors:** Zi-Wei Chen, Satyanarayana M. Chintala, John Bracamontes, Yusuke Sugasawa, Spencer R. Pierce, Balazs R. Varga, Edward H. Smith, Christopher J. Edge, Nicholas P. Franks, Wayland W.L. Cheng, Gustav Akk, Alex S. Evers

**Affiliations:** 1 Department of Anesthesiology, Washington University School of Medicine, St Louis, Missouri, USA; 2 The Taylor Family Institute for Innovative Psychiatric Research Washington University School of Medicine, St Louis, Missouri, USA; 3 Department of Anesthesiology and Pain Medicine, Juntendo University School of Medicine, Tokyo, Japan; 4 Department of Life Sciences, Imperial College, London, UK; 5 UK Dementia Research Institute, Imperial College, London, UK; 6 Department of Developmental Biology, Washington University School of Medicine, St Louis, Missouri, USA

**Keywords:** fluorescence resonance energy transfer (FRET), gamma-amino butyric acid (GABA), ligand binding protein, neurotransmitter receptor, steroid, mass spectrometry (MS)

## Abstract

Propofol is a widely used anesthetic and sedative that acts as a positive allosteric modulator of gamma-aminobutyric acid type A (GABA_A_) receptors. Several potential propofol binding sites that may mediate this effect have been identified using propofol-analogue photoaffinity labeling. Ortho-propofol diazirine (o-PD) labels β-H267, a pore-lining residue, whereas AziPm labels residues β-M286, β-M227, and α-I239 in the two membrane-facing interfaces [β(+)/α(−) and α(+)/β(−)] between α and β subunits. This study used photoaffinity labeling of α_1_β_3_ GABA_A_ receptors to reconcile the apparently conflicting results obtained with AziPm and o-PD labeling, focusing on whether β_3_-H267 identifies specific propofol binding site(s). The results show that propofol, but not AziPm protects β_3_-H267 from labeling by o-PD, whereas both propofol and o-PD protect against AziPm labeling of β_3_-M286, β_3_-M227, and α_1_I239. These data indicate that there are three distinct classes of propofol binding sites, with AziPm binding to two of the classes and o-PD to all three. Analysis of binding stoichiometry using native mass spectrometry in β_3_ homomeric receptors, demonstrated a minimum of five AziPm labeled residues and three o-PD labeled residues per pentamer, suggesting that there are two distinct propofol binding sites per β-subunit. The native mass spectrometry data, coupled with photolabeling performed in the presence of zinc, indicate that the binding site(s) identified by o-PD are adjacent to, but not within the channel pore, since the pore at the 17′ H267 residue can accommodate only one propofol molecule. These data validate the existence of three classes of specific propofol binding sites on α_1_β_3_ GABA_A_ receptors.

The gamma-aminobutyric acid type A (GABA_A_) receptor is considered to be the major molecular target responsible for the anesthetic effect of propofol, the most widely used intravenous anesthetic and sedative in the world. This is supported by the strict correlation of anesthetic activity of propofol analogues with their ability to modulate GABA_A_ receptor currents (1) and by the observation that point mutations in the GABA_A_-β_3_ subunit markedly attenuate propofol anesthesia in mice (2). Propofol can both potentiate currents elicited by low concentrations of GABA and directly activate GABA_A_ currents (3, 4) and functional analysis using the Monod–Changeux–Wyman model indicates that both of these effects are mediated by the same set of propofol binding sites (5).

GABA_A_ receptors are pentameric ion channels composed of varying combinations of 19 subunits (6α, 3β, 3γ, 3ρ, 1δ, 1ε, 1π, and 1θ) each composed of an extracellular domain, a transmembrane domain (TMD) and an intracellular domain. The subunits are arranged in a concentric circle creating an internal ion conducting pore. The TMDs of each subunit are composed of four membrane spanning α-helices (TM1-TM4), with TM2 of each subunit lining the pore and the subunits interacting by contact between TM3 of one subunit (designated the “+” side) and TM1 of an adjacent subunit (designated the “-” side) (6, 7, 8).

Binding sites for propofol on GABA_A_ receptors have been identified using photoaffinity labeling with subsequent protein sequencing by either mass spectrometry (MS) or Edman degradation. The β_3_-H267 residue (17′ on TM2), is labeled by ortho-propofol diazirine (o-PD; Fig. 1*F*) in β_3_-homomeric and α_1_β_3_ GABA_A_ receptors (9). Molecular docking studies indicate a putative propofol binding pocket between TM2 and TM1 of one β_3_ subunit and TM2 of an adjacent subunit (Fig. 1) (10). Based on the location of H267, this binding pocket is referred to as a pore-adjacent site. The functional effects of o-PD on GABA_A_ receptor currents closely replicate those of propofol (9). However, the photochemical products of o-PD preferentially label nucleophilic amino acids such as histidine and may fail to label other residues with which it is proximal. Meta-azipropofol (AziPm; Fig. 1*G*) labels a distinct set of residues in α_1_β_3_ and α_1_β_3_γ_2_ GABA_A_ receptors (β_3_-M286 on TM3, β_3_-M227 on TM1 and α_1_-I239 on TM1) indicating a binding site in the β_3_(+)/α_1_(−) subunit interface and likely binding sites in the α_1_(+)/β_3_(−) and/or γ_2_(+)/β_3_(−) subunit interfaces (Fig. 1) (11). These sites face the lipid bilayer and are referred to as membrane-adjacent sites. AziPm is an excellent photolabeling reagent with no preference for specific amino acids, but poorly replicates the actions of propofol on GABA_A_ receptors: it weakly potentiates GABA-elicited currents and does not directly activate the channel (12).

**Figure 1:**
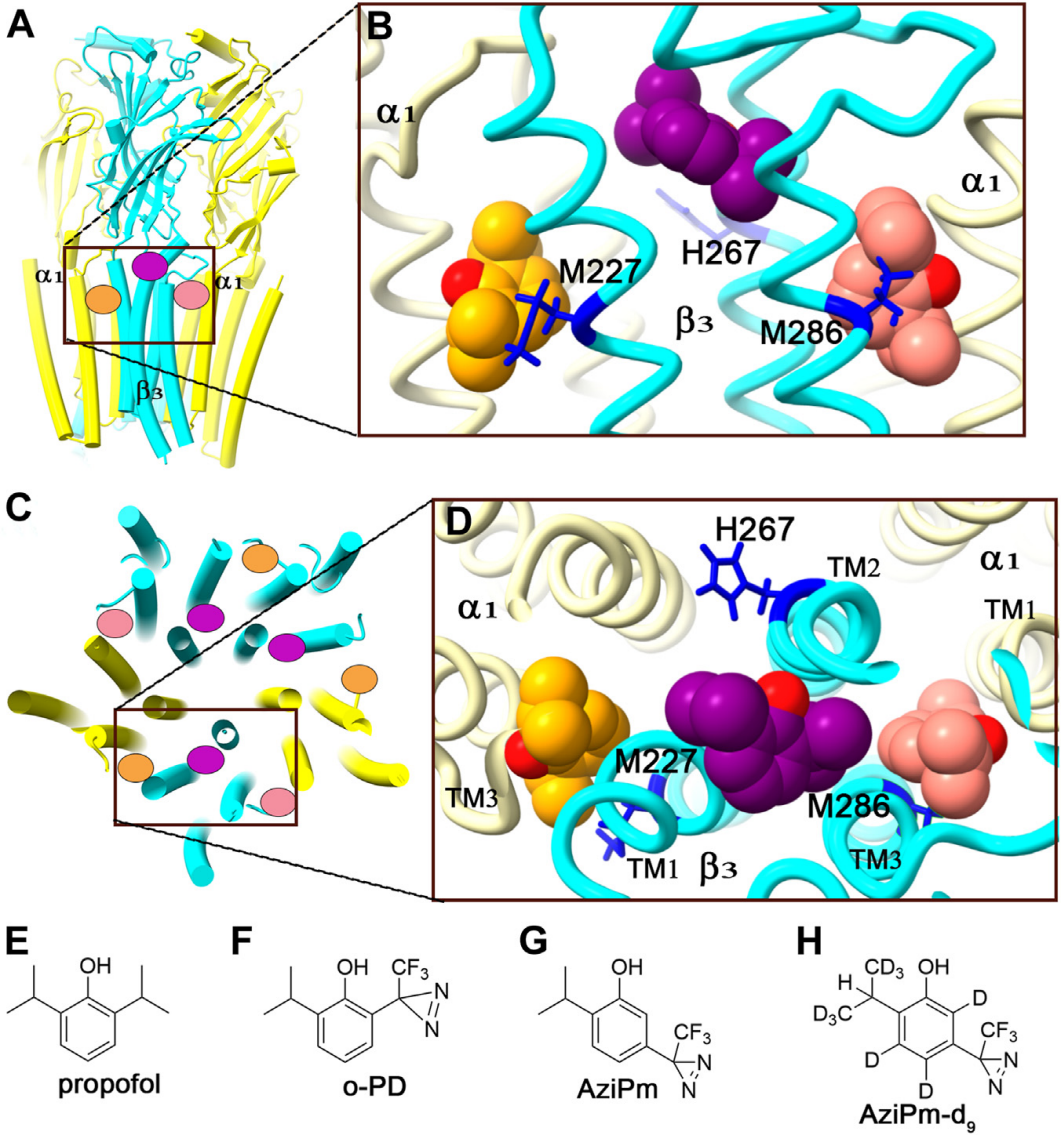
**Propofol binding sites predicted by photolabeling on an α**_**1**_**β**_**3**_**GABA**_**A**_**receptor**. *A*, side view of an α_1_β_3_ GABA_A_ receptor (PDB: 7PBD) with transmembrane domain helices illustrated as *cylinders*. The α_1_ subunits are shown in *yellow* and the β_3_ subunit in *cyan*. Propofol molecules are illustrated as *ovals* with the *orange* propofol in the α_1_(+)/β_3_(−) membrane-adjacent site, the *purple* propofol in the pore-adjacent site and the salmon propofol in the β_3_(+)/α_1_(−) membrane-adjacent site. *B*, magnified side view of binding sites viewed from the membrane face of the receptor with propofol molecules docked in their energetically preferred pose in each binding site. The membrane-adjacent sites are approximately two α-helical turns below the pore-adjacent site. Residues photolabeled by propofol analogues are shown in *stick* format. *C*, vertical view of the α_1_β_3_ GABA_A_ receptor from the extracellular side illustrating that the putative membrane-adjacent propofol binding sites are on the outside of the receptor while the putative pore-adjacent sites are on the pore-facing side. *D*, magnified extracellular vertical view with propofol molecules docked in their energetically preferred pose in each site. *E*–*H*, molecular structures of propofol and the photolabeling analogues o-PD, AziPm, and AziPm-d_9_. GABA_A_, gamma-aminobutyric acid type A; o-PD, ortho-propofol diazirine; PDB, Protein Data Bank.

Site-directed mutagenesis studies provide strong evidence that propofol binding to both the pore- and membrane-adjacent sites predicted by photolabeling are involved in the action of propofol. Tryptophan mutations in the pore-adjacent site (Y143W, F221W, and Q224W) of β_3_-homomeric GABA_A_ receptors markedly reduce direct activation by propofol (13). In α_1_β_3_ receptors, tryptophan mutations in the pore- or membrane-adjacent β_3_(+)/α_1_(−) sites (M286W) reduce propofol potentiation of GABA-elicited currents but do not reduce direct activation. In contrast, double mutations (in both the pore- and membrane-adjacent sites) eliminate potentiation and strongly reduce activation (14). Similar effects of the M286W mutation have been observed with α_1_β_2_γ_2_ receptors (15, 16).

Despite the photolabeling and mutagenesis data, there has been skepticism regarding the existence and functional importance of the pore-adjacent binding site, with recent literature focusing on the β_3_(+)-α_1_(−) intersubunit site as the sole or dominant site for propofol binding and effect (17, 18). Skepticism about the pore-adjacent site arises from the absence of evidence that excess propofol prevents o-PD labeling (9, 11) and from the minimal effect of H267 mutations on propofol action (13, 19). Support for the view that the β_3_(+)/α_1_(−) site is the sole site is enhanced by the demonstration of a propofol density in this site, but not in the pore-adjacent or membrane-adjacent α_1_(+)/β_3_(−) sites, in a cryogenic electron microscopy density map of the α_1_β_2_γ_2_ receptor (17). The current study revisits propofol binding sites, providing additional photolabeling data and placing photolabeling and mutagenesis studies in the context of more recent structural studies. We provide new evidence for the existence and specificity of the pore-adjacent site and show that o-PD binds to both the pore- and membrane-adjacent sites. o-PD does not label the membrane-adjacent sites, likely due to its photochemical specificity. The data also demonstrate that AziPm does not bind to the pore-adjacent site and thus labels only the membrane-adjacent propofol sites. This provides a potential explanation for the inability of AziPm to mimic the effects of propofol on GABA_A_ receptor gating.

## Results

### Prevention of o-PD labeling by propofol and AziPm

To determine whether the β-H267 residue labeled by o-PD identified a specific binding site, we examined the ability of propofol to prevent o-PD labeling. To perform this analysis, we photolabeled HEK cell membranes containing α_1FLAG-His_β_3_-GABA_A_ receptors, solubilized the receptors in n-dodecyl-beta-maltoside (DDM) and affinity-enriched the GABA_A_ receptors using the FLAG epitope tag (20). We then digested the protein with trypsin and directly analyzed the resulting peptides by LC-MS (without solid phase extraction). This allowed us to obtain the full-length tryptic α-helical (TM1-4) peptides from the α_1_ and β_3_ subunits, enabling direct comparison of the intensity of the labeled and unlabeled peptides. In our previous analyses (9, 21), we used multiple, timed chymotryptic and tryptic digests to sequence the receptor. This produced multiple overlapping peptides with cleavages influenced by the presence of the o-PD adduct, making it difficult to assess the relative abundance of the unlabeled and photolabeled peptide.

**Figure 2:**
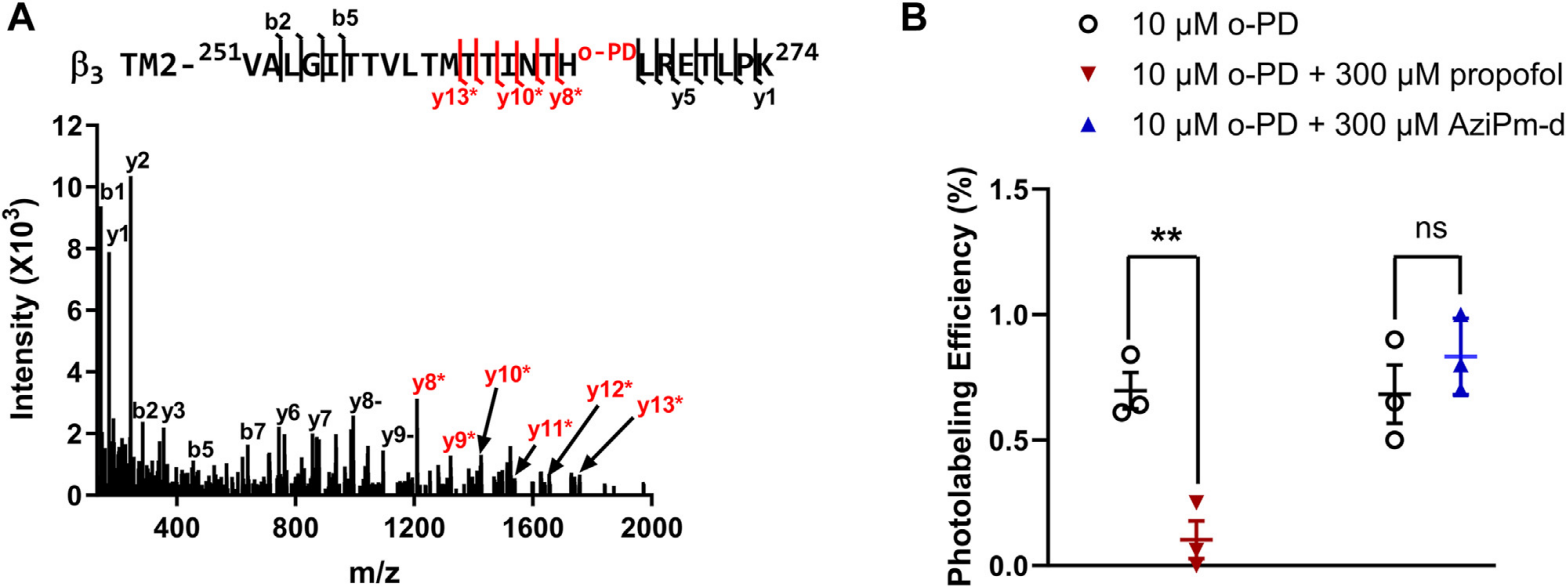
**o-PD photolabeling of the H267 residue on β**_**3**_**-TM2 of an α**_**1**_**β**_**3**_**GABA**_**A**_**receptor is inhibited by propofol, but not AziPm-d**. *A*, fragment ion spectrum of o-PD (10 μM) photolabeling of H267 on the β_3_-TM2 peptide ^251^VALGITTVLTMTTINTHLRETLPK^274^. The y8∗-y13∗ fragment ions (*red*∗) contain the o-PD adduct, whereas the y6 and y7 fragment ions (*black*) do not, indicating H267 as the photolabeled residue. The unlabeled (*black*) y8 and y9 ions represent neutral loss. *B*, photolabeling of the β_3_-TM2 peptide by o-PD (10 μM) in the presence and absence of propofol (300 μM) or AziPm-d (300 μM). Propofol but not AziPm-d prevented o-PD photolabeling. Results were compared using a two-tailed paired *t* test. ∗∗ = *p* < 0.01. GABA_A_, gamma-aminobutyric acid type A; o-PD, ortho-propofol diazirine; TM, transmembrane.

Using this approach, we identified all four of the TM peptides from both the α_1_ and β_3_ subunits of the GABA_A_ receptor with 100% sequence coverage of the TMDs. Two photolabeled peptides were identified: the tryptic TM2 peptide ^251^VALGITTVLTMTTINTH^OPD^LR^269^ (*m/z* = 757.77; *z* = 3) and the same peptide with a single missed cleavage, ^251^VALGITTVLTMTTINTH^OPD^LRETLPK^274^ (*m/z* = 947.191, *z* = 3 and *m/z* = 710.65, *z* = 4), each bearing a single o-PD adduct (mass = 216.08). These peptides met our predetermined criteria for a photolabeled peptide: (1) clearly defined charge states; (2) mass accuracy <20 ppm from theoretical *m/z*; (3) delayed chromatographic retention time (∼2 min) compared to the unlabeled peptide (effect of the hydrophobic adduct); and (4) site-defining fragment ions for the adduct with mass accuracy <20 ppm. The fragment ion spectrum of ^251^VALGITTVLTMTTINTH^OPD^LRETLPK^274^, displayed a series of y ions containing the o-PD adduct (y8-y13, marked in red; mass = 216.08) and a series of y ions (y1–y7) not containing the o-PD adduct (Fig. 2). This spectrum defines the o-PD labeled residue as H267.

Photolabeling with o-PD (10 μM) was then conducted in the presence and absence of 300 μM propofol. The mean labeling efficiency of the TM2 peptides was 0.70 ± 0.07% in control conditions and 0.10 ± 0.08% in the presence of propofol (n = 3; *p* < 0.01; Fig. 2*B*). These data indicate that excess propofol prevents o-PD photolabeling of β_3_-H267, consistent with competitive inhibition of specific binding. We next sought to determine whether AziPm prevents o-PD labeling of H267. AziPm and o-PD have the same molecular mass rendering their photo-adducts indistinguishable by mass spectrometry. To assess whether AziPm prevents o-PD labeling we synthesized the deuterated analogue, AziPm-d (Fig. 1*H*; n.b. AziPm-d is a mixture of the d_6_, d_7_, d_8_, and d_9_ analogues). α_1_β_3_ GABA_A_ receptors were labeled with 10 μM o-PD in the presence and absence of 300 μM of AziPm-d. AziPm-d had no significant effect on o-PD labeling of TM2-H267 (Fig. 2*B*). Even at 300 μM, no AziPm-d-labeled TM2 peptides were identified. However, AziPm-d (300 μM) did label the same peptides/residues in the membrane adjacent sites that are labeled by AziPm (see below). These data indicate that AziPm-d is an effective photolabeling reagent, but does not prevent labeling of the specific binding site identified by o-PD.

### Prevention of AziPm-d labeling by propofol and o-PD

**Figure 3:**
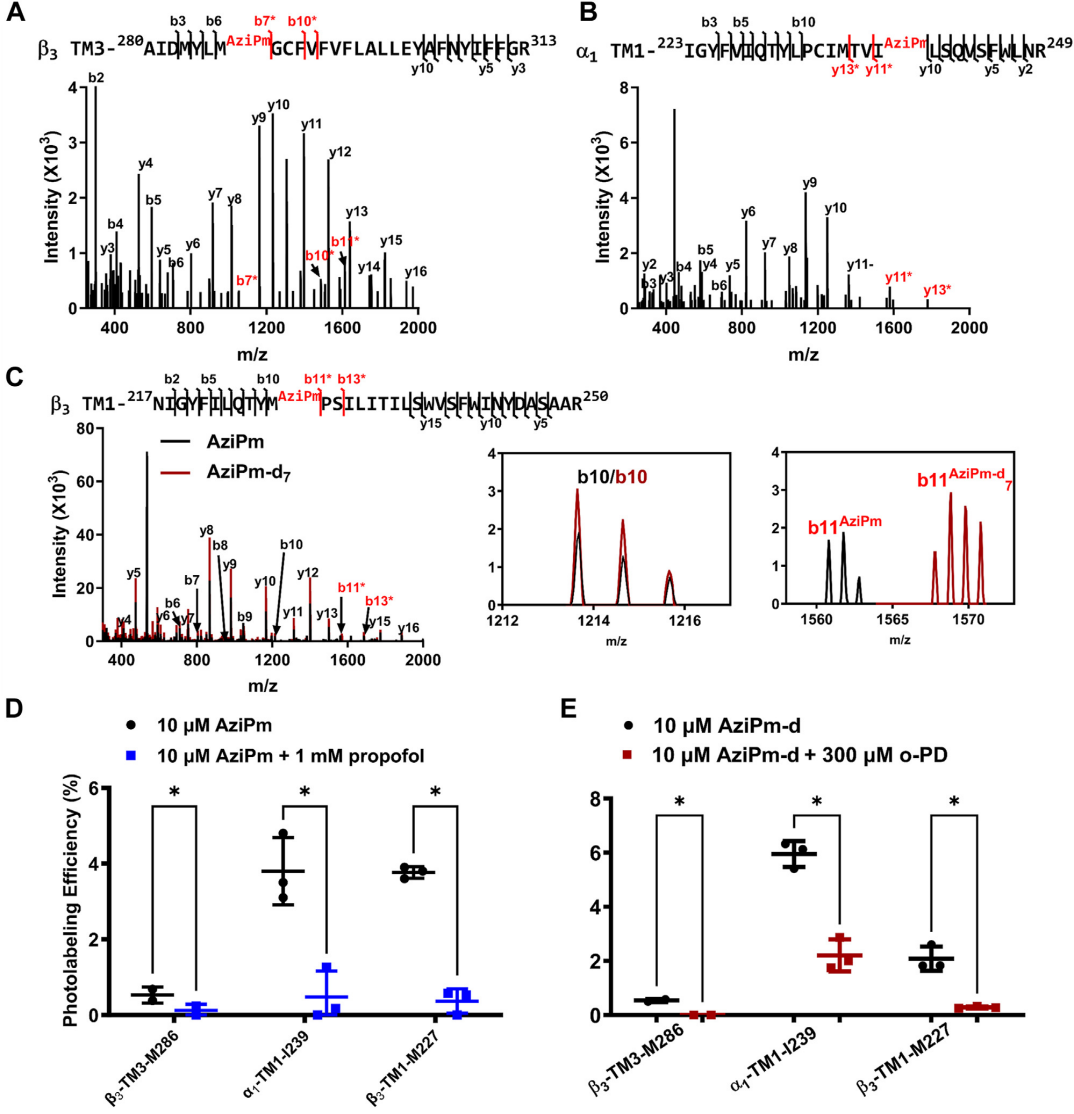
**AziPm photolabeling of membrane-adjacent sites in α**_**1**_**β**_**3**_**GABA**_**A**_**receptors is inhibited by both propofol and o-PD**. *A*, fragment ion spectrum of β_3_-TM3 peptide photolabeled by AziPm on M286 as identified by site defining ions b6 and b7∗ (fragment ions containing the adduct are shown in *red* with a *star*; ions not containing the adduct are shown in *black*). *B*, fragment ion spectrum of α_1_-TM1 photolabeled by AziPm on I239 as identified by site defining ions y10 and y11∗. *C*, overlay of the fragment ion spectra of β_3_-TM1 peptide photolabeled by AziPm and AziPm-d_7_ on M227 as identified by site defining ions b10 and b11∗. The inset illustrates that fragment ions not containing the adduct (b10/b10) are identical in the AziPm and AziPm-d_7_ spectra, whereas fragment ions containing the adduct (b11^AziPm^/b11^AziPm-d7^) differ by 7 da. Precursor ions with an AziPm-d_8_ and AziPm-d_9_ adduct were also detected; in fragmentation spectra of these precursors, fragment ions containing the adduct differed by 8 or 9 mass units from the AziPm adduct, respectively. *D*, photolabeling of β_3_-TM3, α_1_-TM1, and β_3_-TM1 peptides by AziPm is reduced in the presence of propofol (1 mM). *E*, photolabeling of β_3_-TM3, α_1_-TM1, and β_3_-TM1 peptides by AziPm-d is reduced in the presence of o-PD (300 μM). Data in panels *D* and *E* were analyzed using a paired *t* test. ∗ = *p* < 0.05. GABA_A_, gamma-aminobutyric acid type A; o-PD, ortho-propofol diazirine; TM, transmembrane.

A previous study of AziPm photolabeling of purified α_1_β_3_ GABA_A_ receptors using Edman degradation for sequencing identified three labeled residues: β_3_-TM3-M286, β_3_-TM1-M227, and α_1_-TM1-I239. Photolabeling of all of these peptides was prevented by an excess of propofol (11). We repeated this analysis, photolabeling α_1_β_3_ GABA_A_ receptors with 10 μM AziPm and identifying the labeled peptides/residues using LC-MS as described above. Three GABA_A_ receptor tryptic peptides were observed with AziPm adducts (MW = 216.08), all meeting our predetermined criteria for a photolabeled peptide: (1) A β_3_-TM3 peptide, ^280^AIDMYLM^AziPm^GCNEMFVFVFLALLEYAFVNYIFFGR^313^ (*m/z* = 1305.29, *z* = 3), with an N-ethylmaleimide (NEM) adduct on C288 and an AziPm adduct on M286 (Fig. 3*A*). The photolabeling efficiency was 0.53 ± 0.15% (mean ± SEM), which was reduced to 0.08 ± 0.09% (*p* < 0.05; n = 3) in the presence of 1 mM propofol (Fig. 3*D*); (2) an α_1_-TM1 peptide ^223^IGYFVIQTYLPCMTVI^AziPm^LSQVSFWLNR^249^ (*m/z* = 1140.959, *z* = 3) with an AziPm adduct on I239 (Fig. 3*B*). The photolabeling efficiency was 3.80 ± 0.69% which was reduced to 0.47 ± 0.44% in the presence of 1 mM propofol (*p* < 0.01; n = 3) (Fig. 3*D*); and (3) a β_3_-TM1 peptide, ^217^NIGYFILQTYM^AziPm^PSILITILSWVSFWINYDASAAR^250^ (*m/z* = 1046.583, *z* = 4) with an AziPm adduct on M227 (Fig. 3*C*). The photolabeling efficiency was 3.70 ± 0.08% which was reduced to 0.37 ± 0.02% in the presence of 1 mM propofol (*p* < 0.01; n = 3) (Fig. 3*D*). No photolabeling of β_3_-TM2 was observed. These results replicate the AziPm labeled peptides and residues identified using Edman degradation and validate the comparability of the methods.

To investigate whether o-PD prevents AziPm binding, we labeled α_1_β_3_ GABA_A_ receptors with 10 μM AziPm-d in the presence and absence of 300 μM o-PD. AziPm-d labeled the same three peptides and localized to the same residues as AziPm with similar labeling efficiency (Fig. 3*E*). Overlay of the fragment ion spectra from the AziPm and AziPm-d labeled peptides provided additional data for adduct site localization and confirmed that both ligands formed adducts with the same residues. This is illustrated for the AziPM and AziPm-d_7_ labeled β_3_-TM3 peptide, ^280^AIDMYLM^AziPm^GCNEMFVFVFLALLEYAFVNYIFFGR^313^ (Fig. 3*C*) in which fragment ions containing the AziPm adduct (*e.g.* b11) differ by seven mass units between the heavy (AziPm-d_7_) and light AziPM, whereas fragment ions not containing the adduct (*e.g.* b10) have identical *m/z*. (n.b. TM2 peptides containing AziPm-d_6_, -d_8_, and d_9_-labeled peptides were also detected and the fragment ions containing the adduct differed from the AziPm-containing fragment ions by 6, 8, and 9 mass units, respectively.) Both propofol (1 mM) and o-PD (300 μM) significantly reduced AziPm photolabeling efficiency of all three labeled peptides (Fig. 3, *D* and *E*). These data indicate that o-PD prevents AziPm labeling, consistent with o-PD binding to these sites.

### Modulation of GABA_A_ receptor currents by o-PD, AziPm, and propofol

To explore the relationship between differential binding of o-PD and AziPm to pore-adjacent *versus* membrane-adjacent propofol binding sites and their ability to modulate GABA_A_ receptors, we compared the ability of propofol, o-PD, and AziPm to potentiate GABA-elicited currents and to directly activate currents in α_1_β_3_ GABA_A_ receptors expressed in *Xenopus* oocytes. For these experiments, currents are expressed as the percentage (%) of the response to a saturating (30 μM) concentration of GABA.

To assess the ability of the propofol analogues to directly activate GABA_A_ receptors, the receptors were exposed to high concentrations (100 μM) of propofol, o-PD, and AziPm. Propofol and o-PD produced strong direct activation (P_o_ values of 92 ± 17% and 88 ± 4%, respectively), whereas AziPm showed minimal direct activation (P_o_ ∼1%) (Table 1). To assess the ability of the propofol analogues to potentiate the effects of GABA, GABA was first applied at concentrations that produced 7 to 9% P_o_, followed by application of 1, 10, or 50 μM concentrations of propofol, o-PD, and AziPm. Propofol and o-PD strongly potentiated GABA-elicited currents (maximal P_o_ values of 95 ± 7% and 92 ± 2%, respectively) with apparent EC_50_ values between 1 and 10 μM (Table 1). In contrast, AziPm potentiated GABA-elicited currents less potently and likely with less efficacy. A P_o_ value of only 33 ± 14% was achieved with GABA plus 50 μM AziPm (Table 1).

**Table 1:** Direct activation and potentiation of GABA-elicited currents in α_1_β_3_ GABA_A_ receptors by propofol analogues

Ligand	Direct activation by 100 μM	Low GABA (0.1–0.3 μM)	Low GABA + 1 μM	Low GABA + 10 μM	Low GABA + 50 μM
Propofol	92 ± 17%	9 ± 4%	22 ± 12%	78 ± 15%	95 ± 7%
AziPm	1.03 ± 0.95%	7 ± 2%	6 ± 1%	14 ± 7%	33 ± 14%
o-PD	88 ± 4%	7 ± 3%	31 ± 9%	85 ± 6%	92 ± 2%

The α_1_β_3_ GABA_A_ receptors were activated with the indicated concentrations of propofol, AziPm, and o-PD in the presence or absence of GABA. Currents are expressed as the percentage (%) of the response to a saturating concentration (30 μM) GABA. n = 5 for each condition.

### Computational docking of propofol, o-PD, and AziPm to the pore- and membrane-adjacent propofol binding sites

**Figure 4:**
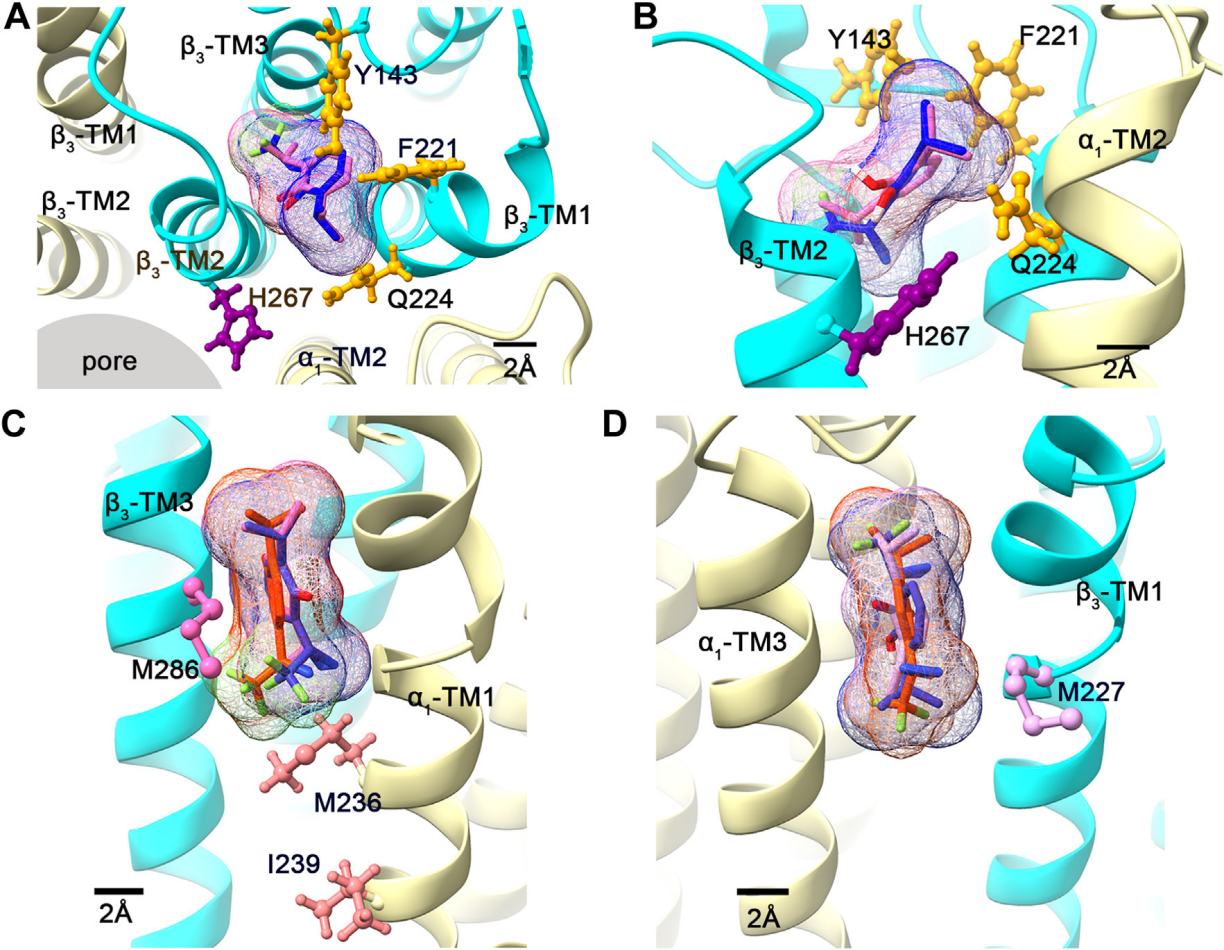
**Computational docking of propofol, o-PD, and AziPM in the pore-adjacent and membrane-adjacent sites in an α**_**1**_**β**_**3**_**GABA**_**A**_**cryogenic electron microscopy structure (PDB:****7PBD****)**. *A*, top-down view (from an extracellular perspective) showing the most energetically favorable poses of propofol (*pink*) and o-PD (*blue*) docked in the pore-adjacent site. The α_1_ subunit is shown in *beige* and the β_3_ subunit in *cyan*. Residues β_3_-Y143, -F221, and -Q224 in the binding pocket are shown in *yellow stick* and *ball* format; the H267 residue photolabeled by o-PD is shown in *purple*. AziPm is not shown because it did not dock in this site. *B*, side view (from membrane perspective) of propofol and o-PD docked in the pore-adjacent site. *C* and *D*, side views (from the membrane perspective) of propofol (*pink*), o-PD (*blue*), and AziPm (*orange*) docked in their most energetically favorable poses in the: (*C*) β_3_(+)/α_1_(−) and; (*D*) α_1_(+)/β_3_(−) membrane-adjacent sites. Photolabeled residues are shown in stick and ball format. GABA_A_, gamma-aminobutyric acid type A; o-PD, ortho-propofol diazirine; PDB, Protein Data Bank.

To identify the energetically favored poses of propofol, o-PD, and AziPm in the three sites identified by photolabeling, we performed *in vacuo* docking to the cryo-EM structure of an α_1_β_3_ GABA_A_ receptor structure (Protein Data Bank (PDB): 7PBD), allowing free rotation of the rotamers of the ligands and the side chains of residues in the putative binding pockets. This approach has two potential limitations. First, we used the structure of a GABA_A_ receptor in a desensitized state for docking because a structure of a GABA_A_ receptor in an open-state conformation, to which propofol should preferentially bind, has not yet been solved. Second, additional information about ligand access and binding might be obtained from molecular dynamic simulation of binding to a GABA receptor embedded in a lipid bilayer. The docking studies show that in the pore-adjacent site, propofol and o-PD exhibit overlapping poses in the pocket between β_3_-TM1, -TM2, and -TM3 and adjacent to α_1_-TM2. The most energetically preferred poses for each ligand are depicted in Fig. 4, *A* and *B*. Propofol and o-PD are identically oriented in a cavity defined by β_3_-Y143, F221, and Q224 (in yellow), adjacent to the TM2 domain of α_1_ at the α_1_(+)/β_3_(−) interface. These amino acids have been previously identified as contributing to propofol potentiation of GABA_A_ currents in site-directed mutagenesis studies using electrophysiological readout (13). The diazirine group of o-PD is situated either 9.2 Å or 8.4 Å from the τ-nitrogen on the imidazole ring of H267 (depending on the orientation of the ortho substituents of o-PD in the binding site), suggesting that H267 may not directly interact with propofol in the binding cavity. Unlike propofol and o-PD, AziPm did not dock in this site. When we attempted to position AziPm in the binding pocket with its hydroxyl group and isopropyl groups in the same position as propofol, the meta-TPD group of AziPm sterically clashes with either β_3_-F221 on TM1 or β_3_-I281 on TM3, depending on which of the propofol isopropyl groups AziPm is aligned with. This provides a plausible explanation for the inability of AziPm to bind in the pore-adjacent site.

In the membrane-adjacent β_3_(+)/α_1_(−) interface site, the most energetically favorable poses for propofol, o-PD and AziPm overlap in position and orientation (Fig. 4*C*). The ligands assume an orientation similar to that observed for propofol in an α_1_β_3_γ_2_ GABA_A_ receptor cryo-EM structure (PDB: 6X3T) (17), with the aromatic ring of propofol facing the M286 side chain and the ortho and meta substituents arranged vertically in the β_3_(+)/α_1_(−) interface. The diazirine moieties of o-PD and AziPm and one of the isopropyl groups of propofol are positioned just above the side chain of M236. The diazirine groups of both o-PD and AziPm are within 6 Å of the backbone α-carbon of β_3_-M286 and α_1_-M236. α_1_-I239, the residue photolabeled with the highest efficiency by AziPm (Fig. 3, *E* and *F*), is oriented toward the same interface as β_3_-M236 but positioned one α-helical turn below M236; the I239 backbone is positioned 11 Å away from the docked position of the AziPm diazirine group.

In the α_1_(+)/β_3_(−) membrane-adjacent site (near β_3_-M227), the energetically preferred poses for propofol, o-PD, and AziPm overlap closely in their position in the interface (Fig. 4*D*). The phenyl groups of all three ligands are parallel to α1-TM3 and β3-TM1 helices with the hydroxyl group pointed into the protein toward TM2. The ortho and meta side chains are oriented vertically in the interface with the diazirine groups of o-PD and AziPm oriented in opposite directions. The diazirine group of AziPm is positioned ∼6 Å away from the backbone α-carbon of β_3_-M227. These docking results support the finding that o-PD binds to the two membrane-adjacent propofol binding sites labeled by AziPm. When an open-state structure becomes available, molecular dynamic simulations of propofol, o-PD, and AziPm binding to an open-state structure should provide more definitive information regarding the molecular details of propofol (and propofol analogue) binding to all of its sites on the GABA_A_ receptor.

### Stoichiometry of o-PD and AziPm labeling of β_3_-homomeric GABA_A_ receptors determined by native mass spectrometry

**Figure 5:**
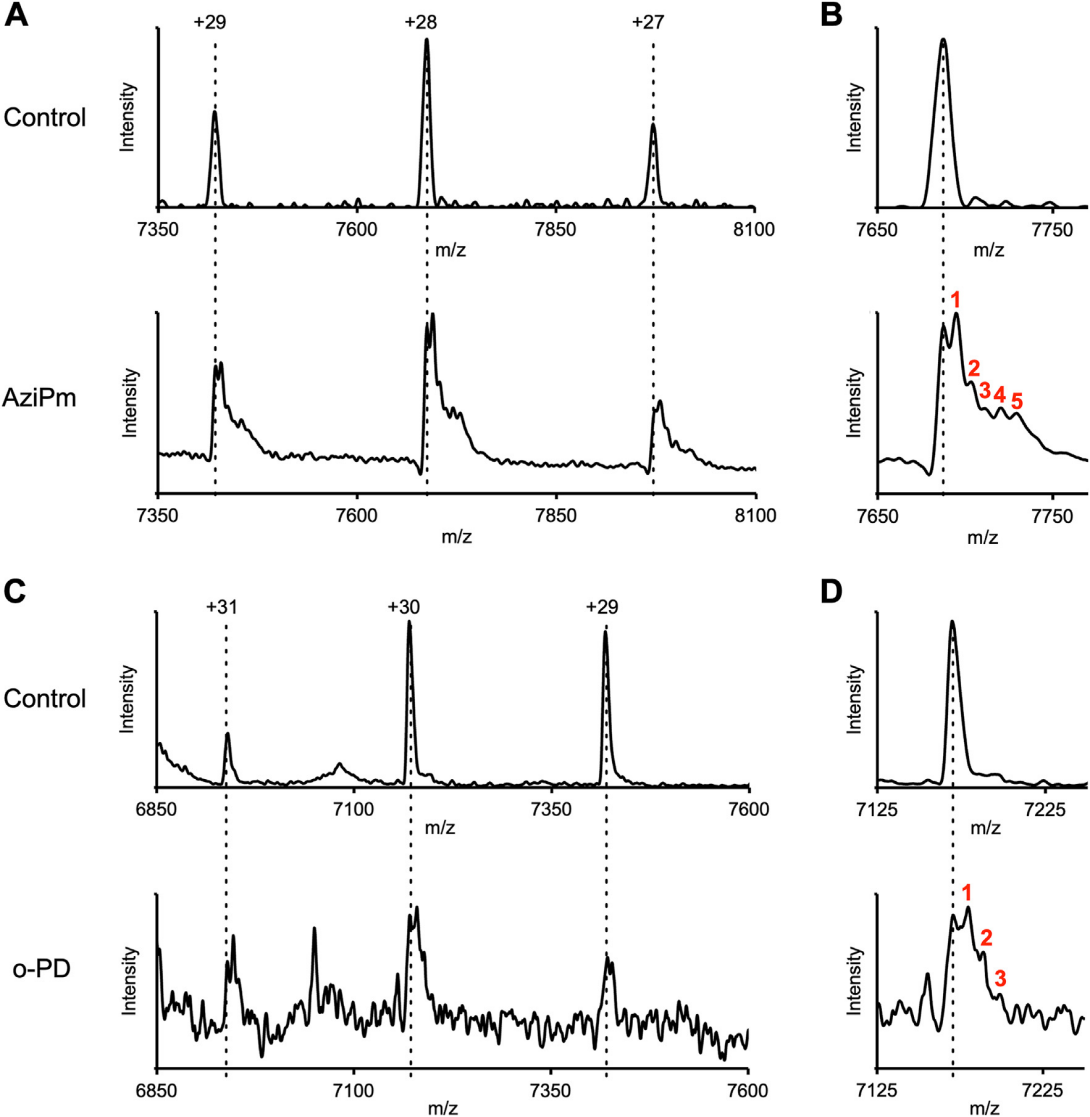
**Native mass spectrometric analysis of photolabeling stoichiometry in homomeric β**_**3**_**GABA**_**A**_**receptors**. *A*, AziPm labeling: (*Top* panel) spectrum of unlabeled β_3_-ILC-del-homopentamer showing three charge-states (+27, +28, and +29). The calculated molecular mass of the pentamer is 215 kDa. (*Lower* panel) spectrum of β_3_-homopentamer photolabeled three times with 200 μM AziPm showing charge states +27, +28, and +29. Charge-state +28 shows 6 features, corresponding to the unlabeled receptor (*dashed line*) and five more features each with an additional mass of 216 ± 17 Da, consistent with AziPm adducts. *B*, same spectra as (*A*) focused on charge-state +28. The *dashed line* indicates the unlabeled β_3_-homopentamer and the numbers in *red* indicate the number of AziPm adducts in a β_3_-homopentamer.*C*, o-PD labeling: (*Top* Panel) spectrum of unlabeled β_3_-ICL-del homopentamer showing three charge-states (+29, +30, and +31) each corresponding to a molecular mass of 215 kDa. (*Lower* panel) spectrum of β_3_-ICL-del homopentamer photolabeled three times with 100 μM o-PD showing charge-states +29, +30, and + 31. Charge-state +30 shows four features, corresponding to the unlabeled receptor (*dashed line*) and three more features each with an additional mass of 232 ± 42 Da, consistent with o-PD adducts. *D*, same spectra as (*C*) focused on charge-state +30. The *dashed line* indicates the unlabeled β_3_-homopentamer and the numbers in *red* indicate the number of o-PD adducts in a β_3_-homopentamer. GABA_A_, gamma-aminobutyric acid type A; o-PD, ortho-propofol diazirine.

β_3_-H267 is located at the 17′ position in the pore of the GABA_A_ receptor and its imidazole side chain can be rotated either into the pore, or toward the adjacent TM2 along the wall of the pore (22). In principle, o-PD could label H267 either from the pore or by entering a pore-adjacent binding site and accessing H267 from within this pocket. Since the diameter of the pore at the 17′ position is ∼9 Å and the cross-sectional area of a propofol molecule is 8.5 Å, the pore can only accommodate a single propofol and a photolabeling reagent in the pore could only label a single H267 per pentamer. If o-PD accesses H267 from a pocket located in each subunit, it could in principle label all of the H267 residues in a GABA_A_ pentamer (2–5 depending on the number of β-subunits in the pentamer). To determine the stoichiometry of o-PD and AziPm photolabeling of a GABA_A_ receptor, we repeatedly labeled the receptors with high concentrations of photolabeling reagent (100 μM X 3 for o-PD, 200 μM X 3 for AziPm) and analyzed the number of covalent adducts on the pentamer using native mass spectrometry. We used purified β_3_-homomeric receptors for these experiments to maximize the potential number of pore-adjacent sites. The spectra for the unlabeled homomeric β_3_-GABA_A_ receptors (upper panels Fig. 5, *A* and *C*) show a single species with narrow charge state distribution (+27 to +29 and + 29 to +31) corresponding to an average mass of 215 kDa. This mass is consistent with a β_3_-GABA_A_ receptor, which includes the calculated mass of the β_3_-ICL-del subunit construct (40.38 X 5 = 201.93 kDa) plus the weight of three Man5-GlcNac2 glycans per subunit obtained in GNTI^-^ cells (∼13.5 kDa) (23). The spectrum of the AziPm-labeled receptors had a significantly broader feature for each charge state (Fig. 5*A*, lower panel) than the unlabeled receptor. Examination of the +28-charge state (the highest intensity charge state) shows a peak corresponding to the unlabeled pentamer plus five more peaks each with an average additional mass of 216 ± 17 Da (shown in red in Fig. 5*B*), consistent with the calculated adduct mass (216 Da) of AziPm. This result indicates a labeling stoichiometry of at least five sites per pentameric receptor. A spectrum of o-PD-labeled β_3_ homomeric receptors also showed a broader feature than the unlabeled receptor (Fig. 5*C*, lower *versus* upper panel). This spectrum showed lower signal-to-noise than the AziPm spectra, possibly due to an effect of the excess o-PD ligand on electrospray ionization. Nevertheless, the +30-charge state (the highest intensity charge state) shows a peak corresponding to the mass of the unlabeled receptor plus three more peaks with an average additional mass of 232 ± 42, consistent with the theoretical o-PD adduct mass (216 Da). These data indicate a minimum o-PD labeling stoichiometry of three sites per pentamer. The labeling stoichiometry of three per pentamer is likely a reflection of the maximum labeling efficiency that can be achieved, limited by o-PD concentration, rather than the maximum binding stoichiometry.

### Zinc inhibition of o-PD photolabeling of β_3_-H267

To confirm that o-PD binds in multiple pore-adjacent propofol binding sites, we exploited the observation that zinc (Zn^2+^) acts as a channel blocker for α_1_β_3_ and β_3_ homomeric GABA_A_ receptors by forming a ternary complex with three H267 molecules at the 17′ position in the pore (24). H267 imidazole side chains complexed to Zn^2+^ in the center of the pore should be inaccessible to labeling by o-PD located in pore-adjacent propofol binding pockets. Zn^2+^ should thus prevent labeling of Zn^2+^-complexed H267 residues. For α_1_β_3_ receptors, all three of the β_3_-H267 residues are coordinated by Zn^2+^ which should thus completely prevent o-PD photolabeling. In contrast, for β_3_-homomeric receptors, only three (or possibly four) of the five β_3_-H267 residues should be coordinated by Zn^2+^ (25) which should thus inhibit o-PD photolabeling by ∼60 to 80%. To test these predictions we photolabeled HEK cell membranes containing either α_1_β_3_ or β_3_-homomeric GABA_A_ receptors with o-PD in the presence or absence of 100 μM Zn^2+^, a concentration that completely blocks current in both types of receptors (26, 27).

**Figure 6:**
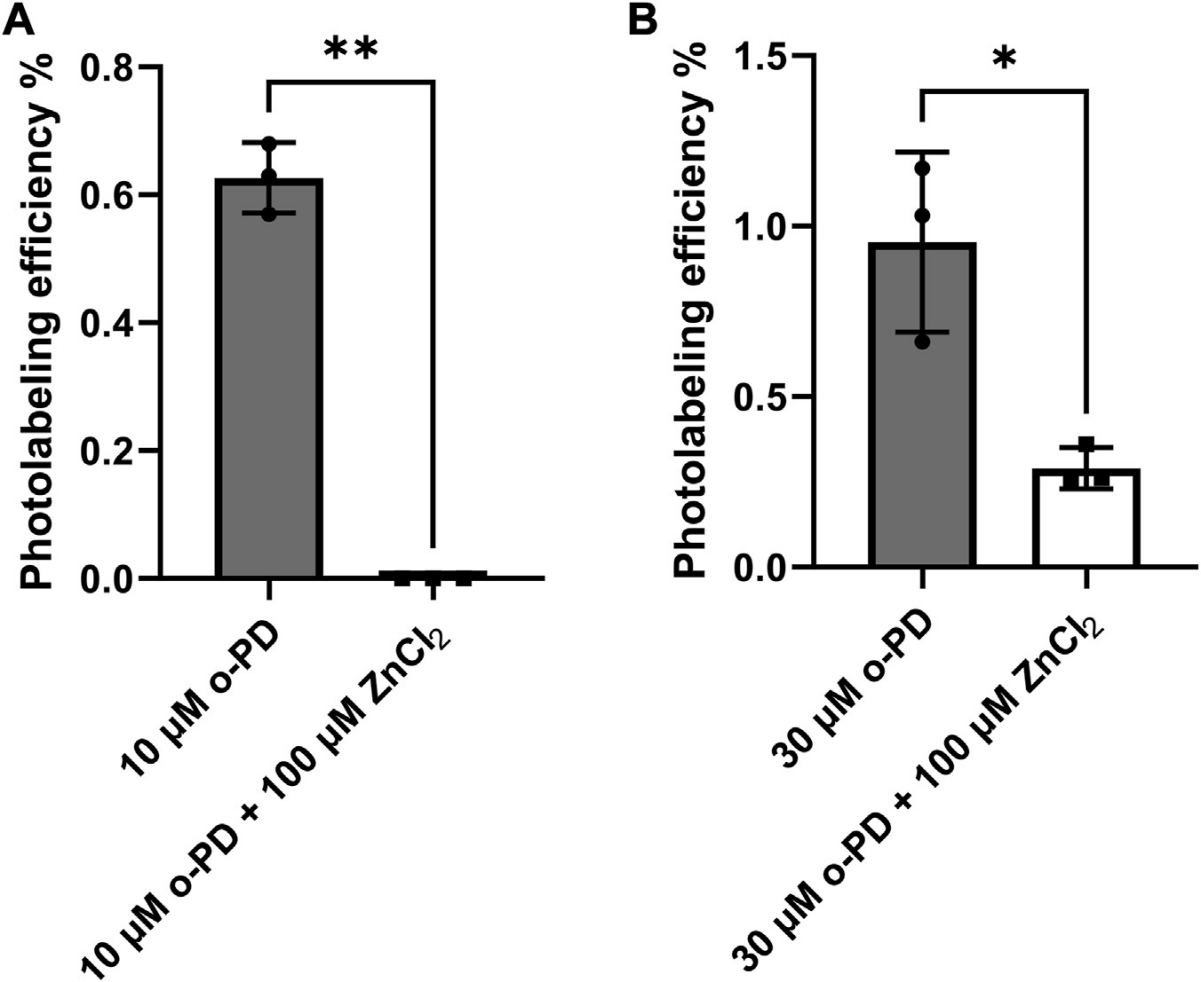
**The effect of ZnCl**_**2**_**on o-PD photolabeling of β**_**3**_**-TM2 peptide in α**_**1**_**β**_**3**_**β**_**3**_**-BRILhomomeric GABA**_**A**_**receptors**. *A*, ZnCl_2_ completely inhibits o-PD photolabeling of β_3_-TM2 peptide in α_1_β_3_ GABA_A_ receptors. *B*, ZnCl_2_ partially (∼70%) inhibits o-PD photolabeling of β_3_-TM2 peptide in β_3_-homomeric GABA_A_ receptors. α_1_β_3_ GABA_A_ receptors were labeled with 10 μM o-PD and β_3_-homomeric receptors with 30 μM to achieve similar photolabeling efficiency. Samples with and without ZnCl_2_ (n = 3 for each) were compared using a unpaired *t* test with ∗ = *p* < 0.05. GABA_A_, gamma-aminobutyric acid type A; o-PD, ortho-propofol diazirine; TM, transmembrane.

HEK cell membranes containing α_1_β_3_ GABA_A_ receptors were photolabeled with 10 μM o-PD in the presence or absence of Zn^2+^ and the labeling efficiency of TM2-H267 was measured using middle-down mass spectrometry. TM2 was labeled with 0.63 ± 0.03% efficiency in the absence of Zn^2+^ and no photolabeling was detected in the presence of Zn^2+^ (Fig. 6*A*). To achieve a similar control photolabeling efficiency, membranes containing β_3_-homomeric GABA_A_ receptors were photolabeled with 30 μM o-PD. The photolabeling efficiency of H267 was 0.95 ± 0.15%. In the presence of 100 μM ZnCl_2_, the photolabeling efficiency was decreased to 0.29 ± 0.03%, representing ∼70% inhibition of photolabeling. These results, along with the labeling stoichiometry determined by native MS, indicate that o-PD labels multiple H267 residues in a GABA_A_ receptor pentamer. This is consistent with o-PD labeling from a pore-adjacent propofol binding site as opposed to a site within the pore.

## Discussion

The goal of this study was to clarify the location and number of specific propofol binding sites on GABA_A_ receptors. The primary result is the validation and further characterization of a specific propofol binding site adjacent to the 17′ β_3_-H267 residue. The ability of propofol to protect H267 from photolabeling confirms that o-PD labeling identifies a specific propofol binding pocket (Fig. 2). This pocket appears to be ligand-specific, as the propofol analogue AziPM-d neither protects H267 from o-PD labeling nor labels nearby residues (Fig. 2). The photolabeling data are supported by molecular docking studies showing that propofol and o-PD assume energetically favorable poses in a pocket between the TM1, TM2, and TM3 of the β_3_ subunit in an α_1_β_3_ GABA_A_ receptor, whereas AziPm does not (Fig. 4). This binding pocket is likely accessed *via* the pore, based on the channel-lining position of the o-PD photolabeled H267 residue and the absence of an apparent tunnel through which propofol could access this site from the membrane (22).

The ability of o-PD to label multiple H267 residues on a GABA_A_ receptor pentamer (at least 3 on a β_3_-homopentamer) is demonstrated by native MS (Fig. 5), suggesting that there may be a pore-adjacent propofol binding site in each β_3_ subunit. Propofol must access these H267 residues from binding pockets surrounding the pore, since only a single propofol (or o-PD) molecule could be accommodated in the pore at the 17′ position. Consistent with this idea, we found that when Zn^2+^ blocks the channel pore by forming a ternary complex with three H267 residues (24), the residual H267 residues in a β_3_-homopentamer can still be photolabeled by o-PD. This indicates that o-PD labeling of H267 must occur from sites surrounding the pore, since the pore itself is occluded by the Zn^2+^-H267 ternary complex and would not accommodate an o-PD molecule.

The proposition that o-PD labeling of H267 identifies specific propofol binding sites adjacent to the channel pore (9, 10) has been challenged with several arguments in addition to the previously noted absence of propofol protection data. First, the finding that H267A (13) and H267C mutations (19) do not interfere with propofol enhancement of GABA_A_ currents has been cited as evidence that H267 does not line a functionally important binding pocket. As observed in docking studies, the diazirine labeling group of o-PD is about 9 Å away from H267, suggesting it is not a crucial residue for propofol binding. Photolysis of o-PD generates a quinone-methide, a reactive intermediate that preferentially labels histidine and cysteine residues (28, 29). The quinone-methide is sufficiently long-lived that it can diffuse to and label the nearby H267 residue; it must however be concentrated in a binding site proximal to H267, as o-PD does not label any of the numerous water-accessible histidine or cysteine residues on GABA_A_ receptors. In contrast, β_3_ subunit residues Y143, F221, and Q224, which line the pore-adjacent binding pocket markedly reduce propofol enhancement of GABA_A_ currents (13, 14, 16), supporting the proposed model. Second, the inability of propofol to protect H267C from modification by a water-soluble pCMBS regent has been interpreted as indicating that H267 labeling does not report on a propofol binding site (19). H267C lines the channel pore and should be readily modified by a water-soluble pCMBS regent in the pore; propofol in a pore-adjacent binding pocket would not be expected to prevent this modification.

We also confirm the findings of a previous AziPm photolabeling study in which Edman degradation was used for sequencing (11). Our data show that AziPm and AziPM-d both label the previously identified β_3_-M286, α_1_-I239, and β_3_-M227 residues, defining membrane-adjacent propofol binding sites on the lipid face of α_1_β_3_ GABA_A_ receptors in the β(+)/α(−), α(+)/β(−), and possibly β_3_(+)/β_3_(−) subunit interfaces. The mass spectrometric method provided complete sequence coverage of the TMDs of the α_1_ and β_3_ subunits and identified no additional labeled peptides. The additional mass units in AziPm-d (as compared to either AziPm or o-PD) facilitated identification of the labeled residues (Fig. 3*C*) and allowed us to examine the ability of o-PD to protect from AziPm-d labeling. Both propofol and o-PD protected the M286, I239, and M227 residues from photolabeling, consistent with previous results obtained using radiolabeled AziPm (11). Docking studies showed that propofol, AziPm, and o-PD all bind in overlapping poses in the membrane-adjacent β(+)/α(−) and α(+)/β(−) propofol binding sites. Interestingly, the most efficiently labeled residue, I239, is two α-helical turns below the position of either the AziPm diazirine in its docked pose in an α_1_β_3_ GABA_A_ receptor (Fig. 4*C*) or the propofol density in the cryogenic electron microscopy structure of an α_1_β_2_γ_2_ GABA_A_ receptor (17). This suggests that AziPm, may assume two poses in the β(+)/α(−) interface: one overlapping propofol in which it labels M286 and a second below propofol in the interface, in which it labels I239.

Collectively, the photolabeling and protection data indicate that o-PD binds to all of the identified propofol binding sites, whereas AziPM binds to the membrane-adjacent propofol binding sites, but not to the pore-adjacent site. These data highlight the limitations of both photolabeling reagents. o-PD closely mimics the positive allosteric effects of propofol on GABA_A_ currents (Table 1), but due to photochemical specificity, labels only a subset of the sites to which it binds. In contrast, AziPm is an effective photolabeling reagent that can label any carbon-hydrogen bond (and hence can label any amino acid) but binds to only a subset of propofol binding sites and weakly mimics the positive allosteric effects of propofol (Table 1). AziPm is a weak positive allosteric modulator of GABA response in α_1_β_3_ GABA_A_ receptors (Table 1) (12) with lower potency and efficacy than either propofol or o-PD. Moreover, it produces virtually no direct activation of the channels, even at 100 μM. The absence of binding to the pore-adjacent site offers one potential explanation for the low efficacy of AziPm. Alternatively, the low efficacy of AziPm could be a consequence of low efficacy in one or both of the membrane-adjacent sites. It is difficult to distinguish between these two mechanisms, and both may contribute to the low efficacy of AziPm.

How many sites contribute to the positive allosteric modulator effects of propofol? The number of sites to which propofol binds on a GABA_A_ receptor depends on the subunit composition. A β_3_ homomeric receptor has five pore-adjacent β_3_ subunit sites and five membrane-adjacent β_3_(+)/β_3_(−) sites for a total of ten sites per pentamer; an α_1_β_3_ receptor has five membrane-adjacent sites [a mixture of β_3_(+)/α_1_(−), α_1_(+)/β_3_(−) and β_3_(+)/β_3_(−)] and three pore-adjacent β-subunit sites for a total of eight sites per pentamer; and an α_1_β_3_γ_2_ receptor has two β_3_(+)/α_1_(−), one α_1_(+)/β_3_(−) and possibly one γ_2_(+)β_3_(−) membrane-adjacent sites, as well as two pore-adjacent β_3_-subunit sites for a total of five or six propofol binding sites per pentamer.

Which of these binding sites contribute to the positive allosteric effect of propofol? β_3_-homomeric receptors provide the simplest case to study with only two types of sites: pore-adjacent and β_3_(+)/β_3_(−) membrane-adjacent sites. Tryptophan mutations of the Y143, F221, and Q224 residues that line the pore-adjacent site (Fig. 4, *A* and *B*) all markedly reduce propofol, but not pentobarbital activation of currents (13). The Y143W and Q224W mutations preferentially reduce activation by bulky propofol analogues, suggesting steric interference with propofol binding by the tryptophan mutations and indicating a functional contribution of the pore-adjacent sites to propofol action. In α_1_β_3_ GABA_A_ receptors, individual amino acid substitutions in either the pore-adjacent site (Q224W, Y143W, and F221W) or the β_3_(+)/α_1_(−) membrane-adjacent site (M286W) have minimal effect on propofol activation, whereas any combination of mutations in the two sites (*e.g.* Y143W/M286W) virtually eliminates propofol activation (14). These data indicate that both the β_3_(+)/α_1_(−) membrane-adjacent sites and the pore-adjacent sites contribute to propofol activation. Finally, a study in α_1_β_2_γ_2_ receptors used concatemeric β_2_-α_1_-γ_2_ and β_2_-α_1_ constructs with mutations of β_2_-Y143W and/or β_2_-M286W to probe the number of functional propofol binding sites and their energetic contributions to propofol gating (16). The results indicate that the two pore adjacent sites and the two β_2_(+)/α_1_(−) membrane adjacent sites contribute equally and additively to channel activation. Analysis of the data in a Monod–Wyman–Changeux framework was best fit by a model with five or six (rather than four) functionally equivalent propofol sites. The α_1_(+)/β_2_(−) membrane-adjacent site is a logical candidate for the additional functional binding site(s) predicted by this model. While a β_3_-M227W mutation has been reported to have no qualitative effect on propofol activation of α_1_β_3_γ_2_ receptors (30), the effect of mutations in this site have not been fully evaluated.

A cryogenic electron microscopic structure of a desensitized α_1_β_2_γ_2_ GABA_A_ receptor with a propofol density in the β_2_(+)/α_1_(−) membrane-accessible site was recently published (17). Propofol densities were detected in neither the pore-adjacent site nor the α_1_(+)/β_2_(−) membrane-adjacent site. Why was propofol not observed in these sites that have been identified by photolabeling (9, 11) and supported by molecular docking (10) and mutational (13, 14, 16) studies? It should be appreciated that when a receptor is photolabeled in a membrane or detergent, the photolabeling reagent can sample multiple conformations of the receptor. In contrast, structural analyses usually identify a single protein conformation, which may or may not be the preferred conformation for ligand binding. Notably, while several structures of GABA_A_ receptors have been published (17, 31, 32, 33, 34, 35, 36, 37, 38), there has not been a structure of a nondesensitized open receptor. It is plausible that propofol preferentially binds to the pore-adjacent and α_1_(+)/β_3_(−) membrane accessible sites in the open state relative to the desensitized state. Indeed, propofol decreases the rate and extent of GABA_A_ receptor desensitization indicating that it should have state-dependent binding that favors the open state (39, 40). The binding of estradiol to a pore-adjacent binding site on the GABA-ρ1 receptor illustrates the conformation-dependent binding of an allosteric modulator to a pentameric ligand-gated ion channel. Estradiol, an inhibitor of ρ1 currents, is observed in the cryo-EM structures of the resting and preopen conformations of the ρ1, but not in the (post open) desensitized conformation (41) A second reason why the propofol sites identified by photolabeling may not be observed in cryo-Em structures is that propofol is a small molecule with low (μM) affinity for its binding sites and may exhibit multiple binding poses. The β_2_(+)/α_1_(−) membrane-adjacent site has been estimated to have a 2-fold higher affinity for propofol than the pore-adjacent site (16), possibly making it easier to detect. Hopefully, as structural resolution continues to improve, the molecular details and state-dependence of propofol binding in each of its sites will become available.

## Experimental procedures

### Synthesis of o-PD

o-PD was synthesized as previously described (9).

### Synthesis of AziPm and AziPm-d_9_

**Figure 7:**
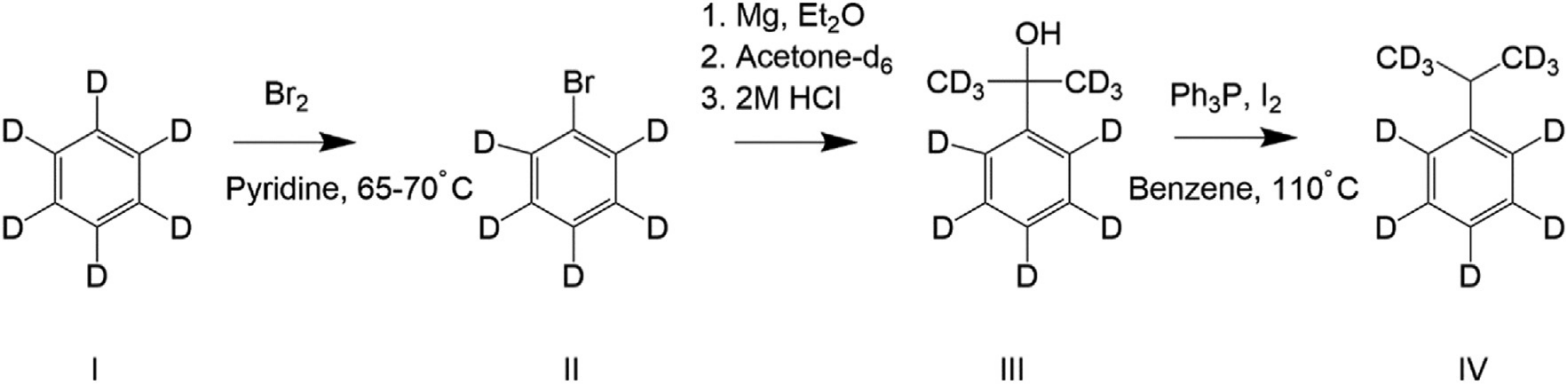
**Synthetic scheme for synthesis of cumene-d**_**11**_**from benzene-d**_**6**_**.**

The synthesis of AziPm was carried out according to the literature method (12). The nona-deuterated version (AziPm-d_9_, I) was synthesized similarly from cumene-d_11_ (IV). This was made, in turn, from benzene-d_6_ (Fig. 7) by way of sequential bromination, Grignard formation, and reaction with acetone-d_6_, followed by reduction with triphenylphosphine/iodine (42).

All reagents and solvents were used as bought from commercial sources. ^1^H, ^13^C, and ^19^F NMR spectra were recorded on a JEOL 400 MHz nuclear magnetic resonance spectrometer. Mass spectra were recorded in either positive or negative mode by electron ionization or electron spray ionization methods. AziPm HRMS negative ion spectroscopy showed *m/z* = 243.0753; *m/z* calculated for [C_11_H_11_F_3_N_2_O-H] = 243.0751.

### Bromobenzene-d_5_

Benzene-d_6_ (25.0 g, 0.297 mol) and pyridine (250 mg 3.16 mmole) were added to a three-necked round-bottomed flask fitted with a magnetic stirrer bar. Bromine (62.4 g, 0.39 mol) was added slowly with delayed evolution of a white gas. After addition of the bromine, the solution was stirred at 40 °C for 30 min followed by 1 h at 70 °C. The contents of the flask were washed with three aliquots of dilute sodium hydroxide solution followed by washing once with water. The organic layer was dried over anhydrous calcium chloride. The liquid was then distilled under water-pump vacuum to give a fraction boiling at 30 to 40 °C. Further distillation under atmospheric pressure gave a fraction boiling at 154 to 158 °C (21.77 g, 45% yield). Electron-ionization MS of the liquid showed two peaks *m/z* = 160.15 and 162.15 *m/z* (Calc. for C_6_D_5_Br (M + H) = 160.9949-, 162.9618).

### 2-Phenyl-2-propanol-d_11_

Bromobenzene-d_5_ (20.9 g; 0.129 mol) was dissolved in dry diethyl ether (10 ml) in a dropping funnel connected to a two-necked round bottom flask equipped with a magnetic stirrer bar and a water-cooled condenser. The flask was charged with magnesium filings (3.8 g; 0.155 mol) and dry diethyl ether (20 ml). A few drops of the bromobenzene-d_5_ solution were added to the mixture and immediate reflux of the diethyl ether was noted. Addition of the remaining bromobenzene-d_5_ was continued at a rate to keep the diethyl ether boiling vigorously. On completion of the addition, the brown solution was stirred for 15 min. Acetone-d_6_ was added at a rate to restart and maintain the boiling of the diethyl ether. The solution was stirred for 2 hours and then poured into 2M hydrochloric acid (75 ml). The organic layer was separated. The aqueous phase was extracted twice with diethyl ether, the organic layers combined and dried over anhydrous magnesium sulfate. The solvent was removed under reduced pressure to give 20 g of an orange liquid which was used without further purification in the next step of the synthesis.

### Cumene-d_11_

The product from the previous step (20 g; 136 mmol) was added with triphenylphosphine (42.9 g; 151.6 mmol), iodine (41.6 g; 163.8 mmol), and benzene (350 ml) into a round-bottom flask equipped with a stirrer bar and water-cooled condenser. The mixture was refluxed for 150 min. After cooling, the mixture was shaken twice with a solution of sodium sulfite to remove iodine. The yellow organic layer was separated and washed once with water. Benzene was removed under reduced pressure to give an orange solid. This solid was then heated under reduced pressure to give two fractions of a light-yellow liquid, one boiling at 56 to 60 °C (F1) and the second boiling at 64 to 84 °C (F2). The two fractions were subjected to proton NMR. F2 showed a peak at 2.9 ppm which is consistent with the desired cumene-d_11_ (2.53 g, 16% yield).

### p-Trifluoroacetylcumene-d_11_ and AziPm-d

An equivalent molar quantity of undeuterated cumene was added to the crude cumene-d_11_ (2.53 g; 19 mmol), thereby allowing a more definitive identification of the mass spectral signal of the AziPm when bound to any given peptide from the GABA_A_ receptor. The mixture was dissolved in dichloromethane (60 ml) and 4-(dimethylamino)pyridine (4.9 g; 40 mmol) was added, followed by trifluoroacetic anhydride (8.76 g; 42 mmol). The solution was cooled to 0 °C in an ice bath followed by addition of anhydrous aluminum chloride (15.9 g; 119 mmol) when the solution slowly turned dark brown. The solution was cooled further to −10 °C in an acetone/ice bath for 15 min after which it was allowed to warm to room temperature and stirred for 5 days. The solution was poured into ice/water (500 ml) and the organic layer separated. The aqueous layer was extracted with two aliquots of dichloromethane, and the organic layers combined and dried over anhydrous sodium sulfate. This was then used without further purification for the continued synthesis of I. A negative ion, high resolution mass spectrum of the AziPm-d product showed features at *m/z* values of 250.1185, 251.1250, 252.1320, and 252.1380 with intensities in a ratio of 21:40:27:12. The calculated *m/z* for AziPm-d_9_ [C_11_H_2_D_9_F_3_N_2_O -H] = 252.1316. These data indicate that the final product is a mixture of AziPm-d_6_, d_7_, d_8_, and d_9_. with AziPm-d_7_ and AziPm-d_8_ being the most abundant species. The reagent is hence referred to as AziPm-d.

### Cell culture, protein expression and membrane preparation

Tetracycline inducible HEK-T-Rex-293 cells stably expressing high-density human α_1_-_8xHis-FLAG_ and human β_3_ GABA_A_ receptor subunits were established as reported previously (20). Briefly, the cells were cultured in Dulbecco's modified Eagle's medium/F-12 50/50 medium containing 10% fetal bovine serum (tetracycline-free, Takara), penicillin (100 units/ml), streptomycin (100 g/ml), blasticidin (2 mg/ml), hygromycin (50 μg/ml), and zeocin (20 μg/ml) at 37 °C in a humidified atmosphere containing 5% CO_2_. Upon reaching 50% confluence, GABA_A_ receptor expression was induced by treating the cells with 1 μg/ml doxycycline with the addition of 5 mM sodium butyrate for 48 to 72 h. After induction, the cells were harvested in a buffer containing 10 mM potassium phosphate, 100 mM potassium chloride (pH 7.5) plus protease inhibitors (Sigma-Aldrich). The cells were collected by centrifugation at 1000*g* at 4 °C for 5 min and homogenized with a glass mortar and a Teflon pestle for ten strokes on ice. Membranes were collected by centrifugation at 40,000*g* at 4 °C for 30 min and resuspended in a buffer containing 10 mM potassium phosphate and 100 mM potassium chloride (pH 7.5). Protein concentration was determined with a micro-bicinchoninic acid protein assay (Thermo Fisher Scientific). GABA_A_ receptor density was determined by measuring the B_max_ of [^3^H]muscimol binding as previously described (20). Membranes proteins were stored at −80 °C.

Two human β_3_ constructs were designed to be expressed using baculovirus in HEK GnTI- cells. The intracellular loop was deleted between residues F307 and A421of the mature peptide and replaced with a short sequence, SQPARAA. Additionally, the 1D4 rhodopsin epitope sequence was added to the complementary DNA (cDNA) to produce a protein with the epitope added to the C terminus for protein purification. This construct has been named hβ_3_-ICL-del 1D4. Another cDNA construct included the BRIL sequence in the intracellular loop region between residues A325 and A421 for the purpose of stabilizing the receptor and is referred to as β_3_-BRIL (24). Both of these genes were synthesized by Twist Bioscience using the pEG BacMam vector designed by Eric Gouaux (43).

Bacmid viral DNA was produced by transforming *Escherichia coli* containing the DH10EMBacVSV bacmid with the cDNA. Using the beta galactosidase blue/white colony selection in the presence of gentamycin, tetracycline, and kanamycin, white colonies were selected that indicated insertion of the cDNA into the transposon elements of the bacmid. These were selected and cultured in LB medium. DNA was prepared from harvested cells, and PCR was done as a quality control to ensure proper insertion and that a full-length gene was present. Bacmid was transfected into Sf9 cells to make the first generation of viral particles (P0). This initial viral culture was introduced into a suspension culture of Sf9 cells (150 ml) with a density of 1 × 10^6^ cells/ml and multiplicity of infection of 0.005. After approximately 6 days, a red color was observed from the coproduction of mcherry fluorescent protein. The viral particles were purified from the Sf9 cells and used to transduce expression in HEK GnTI^-^ cells in suspension at a density of 2 × 10^6^ cells/ml with a multiplicity of infection of 2 on day one. Cells were harvested on day 5, approximately 90 h after induction, by centrifugation at 1000*g*. After removing the supernatant, cells were washed with PBS and resuspended in PBS with protease inhibitors, centrifuged again, and frozen in liquid nitrogen.

### Photolabeling and purification of α_1_β_3_ and β_3_-homomeric GABA_A_ receptors for middle-down MS analysis

For photolabeling experiments, 20 to 40 mg of HEK cell membrane proteins (about 150 pmol of receptor as determined by [^3^H]muscimol binding) were thawed and resuspended in a buffer (10 mM potassium phosphate, 100 mM potassium chloride (pH 7.5), and 1 mM GABA) at a final concentration of 1.25 mg/ml. The membranes were incubated with the photolabeling analogues on ice for 1 h. For experiments examining competitive prevention of photolabeling, membranes were incubated at 4^◦^C for 1 h with o-PD or AziPm-d in the presence or absence of a putative competitor (300 μM propofol, o-PD and AziPm-d_9_, or 100 μM ZnCl_2_); an equal volume of vehicle (ethanol) was added to all samples. The samples were then irradiated in a quartz cuvette for 5 min, using a photoreactor emitting light at >320 nm as previously described (44). The membranes were then collected by centrifugation at 20,000*g* for 45 min at 4 °C. Membrane proteins were solubilized in lysis buffer containing 1% DDM, 0.25% cholesteryl hemisuccinate, 50 mM Tris (pH 7.5), 150 mM NaCl, 2 mM CaCl2, 5 mM KCl, 5 mM MgCl2, 1 mM EDTA, and 10% glycerol at a final concentration of 1 mg/ml. The membrane suspension was homogenized using a glass mortar and a Teflon pestle and incubated at 4 °C overnight. The protein lysate was centrifuged at 20,000*g* for 45 min at 4 °C. The resulting supernatant was incubated for 2 h at 4 °C with 0.2 ml anti-FLAG agarose (Sigma-Aldrich). The anti-FLAG agarose was then transferred to an empty column and washed with 20 ml of washing buffer (50 mM triethylammonium bicarbonate and 0.05% DDM). GABA_A_ receptors were eluted with aliquots of washing buffer containing 200 μg/ml FLAG tag peptide and 100 μg/ml 3X FLAG (ApexBio). The pooled eluates (4 ml) containing GABA_A_ receptors were concentrated to 100 μl using a 100 kDa cut-off centrifugal filter. For homomeric β_3_-GABA_A_ receptor purification, membrane proteins were solubilized in the same lysis buffer used with α_1_β_3_-GABA_A_ receptors and the protein lysate was incubated with Rho-1D4 agarose at 4 °C for 6 h. After washing with 20 ml washing buffer, β_3_-GABA_A_ receptors were eluted with washing buffer containing 500 μM Rho-1D4 peptide at 4 °C overnight.

### Middle-down MS analysis

A middle-down mass spectrometric method was used to selectively detect and sequence the TMDs of GABA_A_ receptors as previously described (20). Affinity-enriched GABA_A_ receptors were reduced with 5 mM tris(2-carboxyethyl)phosphine for 1 h, alkylated with 5 mM NEM for 1 h in the dark, and quenched with 5 mM DTT for 15 min. These three steps were done at room temperature. Samples were then digested with 8 μg of trypsin for 7 days at 4 °C. The digestions were terminated by adding formic acid in a final concentration of 1%, followed directly by LC-MS analysis on an Orbitrap Elite mass spectrometer. Subsequently, 20 μl samples were injected onto a home-packed PLRP-S (Agilent Technologies) column (10 cm × 75 μm, 300 Å), separated with a 145 min gradient from 10% to 90% acetonitrile, and introduced to the mass spectrometer at 800 nl/min with a nanospray source. The survey MS1 scans were acquired at high resolution (60,000 resolution) in the range of *m/z* = 100 to 2000 and the fragmentation spectra were acquired at 15,000 resolution. Data-dependent acquisition of the top 20 MS1 precursors with exclusion of singly charged precursors was set for MS2 scans. Fragmentation was performed using collision-induced dissociation or high-energy dissociation with normalized energy of 35%. The data were acquired and reviewed with Xcalibur 2.2 (Thermo Fisher Scientific).

The LC-MS data were searched against a customized database containing the sequence of the GABA_A_ receptor α_1-8XHis-FLAG_ and β_3_ subunits and filtered with 1% false discovery rate using PEAKS Xpro (Bioinformatics Solutions Inc). Search parameters were set for a precursor mass accuracy of 20 ppm, fragmentation ion accuracy of 0.1 Da, up to three missed cleavages on either side of peptides with trypsin digestion. Methionine oxidation, cysteine alkylation with NEM or DTT, and adducts of o-PD (mass = 216.08), AziPm (mass = 216.08), AziPm-d_7_ (mass = 223.13), or AziPm-d_8_ (mass = 224.13) on any amino acid were included as variable modifications. Photolabeling efficiency was estimated by measuring the areas under the curve (AUC) of selected ion chromatograms of the photolabeled and corresponding unlabeled peptides and calculating the ratio of the labeled peptide AUC/(labeled peptide AUC + unlabeled peptide AUC). In AziPm-d labeling experiments, photolabeling efficiency was estimated using the sum of the AUC for AziPm-d_7_ and AziPm-d_8_ adducts. Results are expressed as percent efficiency. Efficiency of photolabeling between conditions was compared using paired *t* test for binary comparisons (Prism 10, GraphPad Software, San Diego, CA; https://www.graphpad.com/scientific-software/prism/). The MS2 spectra of photolabeled TMD peptides were also manually analyzed for fragment ion charge state and mass accuracy and to confirm the sequence assignment and sites of adduction.

### Native MS of photolabeled β_3_-homomeric GABA_A_ receptors

For native MS experiments, β_3_-ICL-del receptors were solubilized in 1% 2,2-dioctylpropane-1,3-bis-β-D-maltopyranoside (DMNG), affinity-purified on Rho-1D4 antibody-coupled beads followed by further purification by size-exclusion chromatography (22). Subsequently, 30 μg of purified human β_3_-ICL-del GABA_A_ receptor in 75 μl buffer (10 mM Hepes, pH 7.2, 150 mM NaCl, 0.007% (w/v) DMNG, 0.0006% (w/v) cholesteryl hemisuccinate) was preincubated with photolabeling reagent at 4 °C for 30 min prior to UV-irradiation. To achieve maximum photolabeling efficiency, AziPm was added to the protein at a concentration of 200 μM three times with UV-irradiation between each application. o-PD was applied to the sample at a concentration of 100 μM three times with UV-irradiation between each application. A lower concentration of o-PD was necessary to perform native MS analysis. Even at this concentration, the native MS spectrum for the o-PD sample showed more noise, possibly due to a detrimental effect of free o-PD on the electrospray ionization process. The AziPm and o-PD photolabeled samples were prepared from two separate β_3_ GABA_A_ receptor purifications, and nonphotolabeled β_3_ GABA_A_ receptor samples were analyzed in each case. After photolabeling, the samples were buffer exchanged into 200 mM ammonium acetate pH 8 and 0.08 mM DMNG (∼2 times critical micelle concentration [CMC]) using Bio-Spin gel filtration columns (Bio-Rad). Briefly, 3 μl of this sample was directly loaded into a borosilicate capillary emitter (Thermo Fisher Scientific, ES380) and native MS spectra were obtained by static nanospray on a Thermo Q-Exactive EMR mass spectrometer. The data were collected using an electrospray voltage of 1.2 to 1.5 kV, capillary temperature of 200 °C, resolution of 8750, trap and transfer voltages of 200 V (CID) and 150 V (CE), respectively, for the Azi-Pm sample. For the o-PD sample, the transfer voltage was increased to 200 V. Ion transfer optics were set at 8, 7, 6, and 4 V for injection flatapole, interflatapole lens, bent flatapole, and transfer multiple, respectively. Since the o-PD spectrum had significant noise, all spectra were deconvoluted manually using the three observed charge states, and the molecular weight determined by averaging the mass from the three charge states. For the peak corresponding to 2 o-PD labels in the β_3_ GABA_A_ receptor pentamer, only the +30- and +31-charge states were used, and for the peak corresponding to 3 o-PD labels, only the +30-charge state was used. The observed charge states differed between the experiments performed with o-PD and Azi-Pm. This variability is commonly observed and may be a consequence of differences in the protein sample between preparations or variations in the capillary emitter tip structure.

### Docking simulations

The molecular coordinates of propofol, o-PD, and AziPm were generated by Avogadro software (https://avogadro.cc/) with minimized energy and converted into a PDB file. A model of the human α_1_β_3_ GABA_A_ receptor in the presence of GABA (Protein Data Base bank ID: 7PBD) was used for docking. Docking preparation including deletion of water and addition of charges was performed in Autodock tools. All three ligands as well as amino acid residues of GABA_A_ receptors surrounding the photolabeling sites were set as flexible in Autodock tools. The flexible residues for the pore-adjacent site are as follows: β_3_Y143, β_3_Y220, β_3_Q224, β_3_Th225, β_3_P228, β_3_T263, β_3_I264, β_3_H267, β_3_L268, β_3_I281, α_1_T267, α_1_I271, and α_1_N275t; for the membrane-adjacent α_1_(+)/β_3_(−) site: β_3_I222, β_3_L223, β_3_Q224, β_3_M227, β_3_P228, β_3_I230, β_3_L231, α_1_D287, α_1_W288, α_1_A291, α_1_V292, α_1_Y294, and α_1_A295; and for the membrane-adjacent β_3_(+)/α+(−) site: β_3_M283, β_3_M286, β_3_V290, α_1_I228, α_1_L232, α_1_l235, α_1_M236, and α_1_I239. The docking was performed using AutoDock Vina (https://vina.scripps.edu/) (45). Docking grid boxes were built to cover the whole TMD domain with dimensions of 50 × 50 × 45 Å for initial search and 25 × 25 × 25 Å for each individual site encompassing all the flexible amino acid residues. Docking was limited to an energy range of 4 kcal from the best docking pose. Docking poses were visualized in UCSF chimera 1.16 and ChimeraX.

### Receptor expression in *Xenopus laevis* oocytes and electrophysiological recordings

Human α_1_β_3_ GABA_A_ receptors were expressed in oocytes from the African clawed frog (*X. laevis*). Oocyte preparation and complementary RNA injections were done as described in detail previously (46). The complementary RNA injection ratio was 5:1 (α_1_:β_3_, 2.5 ng: 0.5 ng) to reduce the expression of homomeric β_3_ receptors. The electrophysiological recordings were conducted using standard two-electrode voltage clamp. The oocytes were clamped at −60 mV and perfused with ND96 (96 mM NaCl, 2 mM KCl, 1.8 mM CaCl2, 1 mM MgCl_2_, and 5 mM Hepes; pH 7.4) at 5 to 8 ml/min. Solutions were gravity-applied from glass syringes with glass luer slips *via* Teflon tubing. A typical potentiation experiment consisted of exposing an oocyte to 0.2 to 0.3 μM GABA (EC7-9), followed by coapplications of GABA and test compound and, for normalization purposes, an application of 30 μM (saturating) GABA. Direct activation experiments consisted of application of 100 μM test compound followed by application of 30 μM GABA. Drug applications were separated by 2 to 3 min washes in ND96. The current responses were amplified with an OC-725C amplifier (Warner Instruments), digitized with a Digidata 1200 series digitizer (Molecular Devices), and stored using pCLAMP (Molecular Devices; https://www.moleculardevices.com/products/axon-patch-clamp-system/acquisition-and-analysis-software/pclamp-software-suite). The analysis of current traces was done with Clampfit (Molecular Devices). The stock solution of GABA was made in ND96 bath solution at 500 mM, stored in aliquots at −20 °C, and diluted as needed on the day of experiment. The steroids were dissolved in dimethyl sulfoxide at 10 to 20 mM and stored at room temperature.

## Data availability

All the data described in this manuscript is contained within the manuscript. Raw mass spectrometry data files will be shared upon request to the corresponding author (eversa@wustl.edu).

## Conflict of interest

The authors declare that they have no conflicts of interest with the contents of this article.
